# Surviving Psychiatry On-Calls: A Peer-Led Video Intervention

**DOI:** 10.1192/bjo.2026.11371

**Published:** 2026-06-30

**Authors:** Tlhompho Gwen Ditedu, Oriyomi Shittu, Arun Viswanath

**Affiliations:** South West Yorkshire Partnership Teaching NHS Trust, Barnsley, United Kingdom

## Abstract

**Aims:**

A peer-led video intervention aimed at boosting trainee confidence in handling psychiatry on call tasks.

Assess what trainees are most worried about before starting out their psychiatry on-calls.

Assess baseline trainee confidence of handling on call tasks.

Design a targeted video intervention aimed at junior resident doctors (both core and foundation trainees) to boost confidence in handling their on-call requirement and assess its impact on trainee confidence.

**Methods:**

We produced an eight-minute introductory tutorial video for core and foundation trainees for their first on calls at Kendray Hospital in Barnsley, to improve their confidence on handling on-call tasks. This video covered key areas of common tasks such as how to clerk a new patient, how to complete a section 5(2) form or a seclusion review. Kendray Hospital specific information such as where to find the on-call pager, and on-call rooms was also included. Microsoft video editing software (Clipchamp) was used for video editing. A survey was sent to assess trainee confidence before and after watching the video. They were asked to rate their confidence on different aspects of on-call tasks on a Likert scale from 1–5.

**Results:**

50% of core and foundation trainees (5/10) responded. Trainees demonstrated an increase in mean confidence scores (on a scale of 1–5) in a few areas. There was a +0.6 in managing agitated patients, +0.6 points increase in confidence of when and how to request section 17 leave, +0.4 increase in confidence on how to complete section 5(2) paperwork. Confidence increased by +0.2 on knowledge of how to access the on-call room. Areas that showed no improvement in confidence where how to handover during shift changes; how carry out a seclusion review, where to locate the on-call pager or how to contact seniors when on call. Trainees demonstrated high confidence in these areas showing that these areas are well covered in other parts of induction into a new rotation.

**Conclusion:**

A peer-led video intervention that offers trust-specific advice can be a small cost-effective way to boost trainee confidence in carrying out on-call tasks. Benefits can beincreased by embedding the video into the official Barnsley trainee’s induction programme to ensure that every new cohort of new trainees have access to it. It is also recommended that the trust include the video in the trust intranet to allow trainees to have access to it any time they need a refresher.

